# Asthma and Allergy Mobile Apps in 2018

**DOI:** 10.1007/s11882-019-0840-z

**Published:** 2019-02-02

**Authors:** Steve Kagen, Amy Garland

**Affiliations:** The Kagen Allergy Clinic, S.C, Appleton, WI USA

**Keywords:** Digital health, App, Mobile application, Asthma, Allergy, Symptom score test, Peak expiratory flow, Outcome measures, Smartphone, mHealth

## Abstract

**Purpose of Review:**

This paper offers a comprehensive review of interactive mobile allergy and asthma smartphone applications available within the USA in 2018, with an emphasis on interactive asthma apps.

**Recent Findings:**

Primary care and specialty clinicians interested in introducing digital health apps into their practices will soon have more choices, for Apple® and major electronic medical record software companies are investing heavily in the mobile medical marketplace, guaranteeing personal health information and access to care will always be immediately available in one’s *digital hand*.

**Summary:**

Interactive mobile asthma applications are valuable assets for patients and caregivers alike, for they offer immediate communications between patients and those responsible for providing for their needs.

**Electronic supplementary material:**

The online version of this article (10.1007/s11882-019-0840-z) contains supplementary material, which is available to authorized users.

## Introduction

Access to health care today is literally in your hand. For early in 2018, there were 325,000 mobile health apps available to smartphone owners worldwide with an additional 200 health apps being launched daily [1]. We have prepared this review article to help clinicians better understand the many digital health opportunities available to monitor and communicate with their allergy and asthma patients.

Mobile health applications are most often developed to monitor a specific health disorder, to inform users about one’s general health and wellness, or to make it easier to manage one’s medications. Given the plethora of interactive and standalone allergy apps to review, this article will focus upon interactive mobile health apps that are free to consumers in the USA in October 2018, with an emphasis on five well-established asthma apps that offer features most preferred by consumers and caregivers.

Standalone (i.e., non-interactive) allergy and asthma apps that do not transmit health information between patients and caregivers are mentioned herein, but are not discussed in detail. Nor will the exciting interactive wearable medical devices.

Mobile health (mHealth) apps are dynamic digital works of art designed to meet the needs of a specific audience. In essence, interactive mHealth apps are dynamic living art, for their development evolves over time and requires significant investments of time, creativity, and resources using the agile development process of build-measure-learn (BML). After evaluating the needs of a specific market, app developers build the software solution, measure user responses, and learn how to improve the app to best meet marketplace expectations. Consequently, mHealth apps and BML are really big deals.

To succeed, digital health apps must satisfy the perceived needs of patients, for patients must always be at the center of the health care universe. But patients, caregivers, payers, sponsors, and government regulators have different interests and judge mobile medical apps through their own unique points of view. What do patients want in an asthma app? What do physicians and nurses prefer? What criteria will regulators use to determine potential risks to consumers and patients when using a mHealth app? These questions will be discussed herein with objective evaluations of five industry leading interactive asthma apps available in the USA in October 2018.

## Digital Health Marketplace

The marketplace for allergy and asthma apps in the USA includes 16 million adults and 5.5 million children with allergic rhinitis, and 6 million children, and 20 million adults with asthma in 2016 according to the National Center for Health Statistics [2, 3].

Every patient with access to a smartphone may benefit from allergy and asthma apps. Higher costs for smartphones, however, are a significant economic impediment for low-income families, even though this population could benefit most from accessing asthma specialty care via live telemedicine app services.

Digital health enterprises market their health care services nationwide, extending far beyond in-office medical care traditionally provided within vertically integrated clinics and hospitals. Retail pharmacies, grocery store chains, and health insurance companies are offering online medical services to consumers via mobile apps, as they openly compete for patients usually seen in private practices and hospitals with an emphasis on chronic illnesses such as asthma, diabetes, obesity, and depression [4–6].

Indeed, workers who receive employer-sponsored health coverage frequently have access to in-plan telemedicine services at no out-of-pocket cost [7]. As mobile health services continue to expand, telemedicine will begin to disrupt America’s health care delivery system, doing to vertically integrated hospitals and clinics what Amazon did to big box retail stores—deliver quality products and services at affordable prices when and where consumers prefer. During this process, specialists in allergy and asthma must be prepared to embrace telemedicine and digital health technology as integral parts of their new-normal clinical practices [8].

## App Development

Creating a successful allergy and asthma mobile application is a complex and costly adventure. It requires building a team of talented and experienced software developers, smartphone and website designers, social media specialists, business attorneys acquainted with state, federal and international health care regulations, motivated physicians, nurses and respiratory therapists, clinic administrators, hospital decision makers, behavior health experts, psychologists, statisticians, working relationships with electronic health record companies, risk management specialists, selection of a secure Cloud-based online data storage entity, and more. All of which must be patient-centric.

In short, digital health app development involves a merger between art and science, guaranteeing an occasionally frustrating yet always entertaining endeavor.

The initial decision in app creation is to determine the product’s purpose and business model. Will it be for-profit or non-profit? What is its target market: patients, caregivers, payers, insurers, and business owners? Will it involve patient education, medical decisionmaking, disease monitoring, or prescription adherence? Will it be a standalone or interactive app? What features will it include: telemedicine, validated asthma symptom score tests, spirometry, fractional exhaled nitric oxide (FeNO) measurements, secure notifications, and real-time monitoring of asthma control?

Because first impressions last forever, a medical app must be beta tested repeatedly before being publicly released to guarantee its design, usability, reliability, and functions meet marketplace expectations. But developing and sustaining a successful mHealth app is never over. It is a continuous process that employs the repetitive feedback loop of BML and repeat: build a minimally viable product for a specific market, quantitatively measure user responses, learn how best to re-build it to meet market expectations—repeating this process again and again. The concept of BML applies to practicing medicine as well, for a caregiver is only as good as his or her last performance.

## What People Want

### Patients and Caregivers

**Fig. 1:**
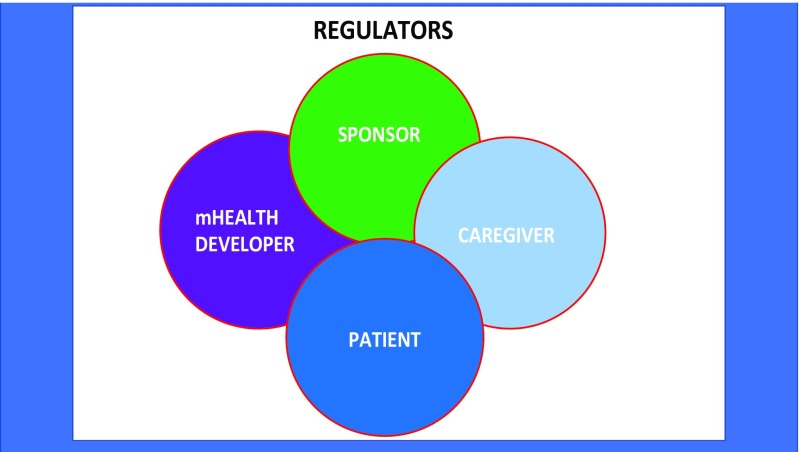
Digital health stakeholders

Selecting an appropriate mHealth app depends upon who you are and what features one is seeking, as well as the app’s quality, price, and services. But every patient, physician, nurse, clinic administrator, insurer, payer, employer, and regulator has their own interests and points of view (Fig. 1). Beauty, like truth, exists in the mind of the beholder.

Surveys of patients and clinicians have revealed that patients are primarily interested in access to educational materials, telemedicine services, and being alerted when their disease is out of control. Caregivers prefer seeing symptom score test results, air pollution and pollen levels, and medication adherence data. Overall, caregivers view mobile health technology as enhancing the patient-centered medical home [9–11]. Pulmonologists prefer obtaining mobile spirometry results between in-office visits, while pediatricians do not share this view. All caregivers, however, are concerned about the accuracy of data obtained and security of patient information.

In collaboration with the Allergy & Asthma Network [12], we surveyed allergy patients and members of the American College of Allergy, Asthma and Immunology (ACAAI) to determine which features they would use in a mobile asthma app. Patients preferred asthma apps that are free, easy to use, accurately monitor symptoms over time, and offer instructions on what to do during asthma exacerbations. Patients were also interested in replacing in-office visits with telemedicine services. Allergy specialists prefer mobile apps that record symptom scores, improve medication adherence, document lung functions, inform patients when to telephone their clinics for help, and alert caregivers when patients are using rescue inhalers more frequently than prescribed (Tables 1 and 2).

**Table 1:** Features preferred by 239 asthma and allergy patients

Feature	% Prefer
Asthma education material	94
Symptom forecast	92
Asthma action plan	92
Telemedicine	90
Connect with local specialists	90
What to do in emergency	90
Monitor symptoms	87
Identify airborne triggers	87
Notifications from clinic	86

Medication adherence was the least preferred feature by patients (83%)

Patient questionnaire: Would you use a mobile app if it:

1. Identifies nearby factors in the air that trigger your symptoms?

2. Provides you with your personal 4-day symptom forecast?

3. Advises you when to call your doctor?

4. Can monitor your symptoms over time?

5. Can show your caregivers how you are feeling?

6. Sends you a warning when your symptoms are out of control?

7. Allows you to receive secure notifications from your doctor’s office?

8. Has an Asthma Action Plan to help you if your asthma worsens?

9. Has educational materials about allergy and asthma?

10. Tells you what to do during a medical emergency?

11. Records your side effects from taking medications or other treatments?

12. Detects if your symptoms are better after you take a new medication?

13. Enables you to see your doctor in live Telemedicine video calls?

14. Connects you with nearby certified specialists in allergy and asthma?

15. Records when you use your inhaled medications?

**Table 2:** Features preferred by 114 asthma specialists

Feature	% Prefer
Symptom score tests	89
Air pollution	89
Pollen and mold information	82
Adherence with medications	81
Weather factors	79
Sleep patterns	73

Diet, emotional stress, heart rate, fractional exhaled nitric oxide levels (FeNO), and spirometry were the least preferred features by physicians (54–63%)

Allergy and asthma specialist questionnaire: Which environmental factors and body measurements would you like to see in a mobile app?

1. Air pollution (ozone, particulate matter)

2. Allergens (pollens, mold spores)

3. Weather factors (temperature, humidity, wind)

4. Peak flow test results

5. Airway inflammation (FeNO) test results

6. Coughing episodes

7. Heart rate and activity levels

8. Emotional stress levels

9. When patients use their inhalers

10. What my patients eat

11. Sleep patterns

12. Symptom score test results (rhinitis, asthma)

13. Other factors or triggers: results of spirometry (FEV-1)

### Insurers and Employers

Life and health insurance corporations believe in the potential health benefits of mHealth and have established reward programs that offer annual insurance premium discounts for using wearable devices, including the Apple Watch and Fitbit [13].

Mobile asthma apps are also being used by health insurers to determine if prescribed treatments are cost-effective and to compete directly with hospitals, medical clinics, and private practitioners. Using an insurance corporation’s smartphone app, or Internet website portal, employees who receive health care benefits at work may choose to *see* an employer-paid physician via telemedicine often at no out-of-pocket cost, creating a potential conflict of interest, for such physicians are servants of the insurance company—not the patient [14].

### Regulators

The US Food and Drug Administration (FDA) and Federal Trade Commission (FTC) play central roles in guiding the development and regulation of mobile medical devices [15–20]. Entities interested in creating mHealth apps must become familiar with existing FDA rules, regulations, and guidance. As a consequence of the recently enacted amendment to the Twenty-First Century Cures Act, the Food, Drug and Cosmetic Act, some software functions are no longer considered medical *devices* [17] Therefore, the FDA is now using enforcement discretion for mobile medical apps that do not present risks to patients and consumers. Importantly, developers of mobile apps and medical software are not required to submit their products for premarket reviews nor register them with the FDA. Practical examples of mHealth apps that do not require FDA reviews can be found online [21].

To protect patients and consumers, the FDA is building a new regulatory model to assess the safety and effectiveness of software technologies without inhibiting consumer access to new technology. The FDA’s new Digital Health Software Precertification (Pre-Cert) Program is now reviewing feedback from app stakeholders as the FDA begins to form its consumer friendly, flexible, and adaptive regulatory framework [18]. The rapidly expanding mHealth marketplace will benefit from common sense regulations that can be modified and improved over time, as will software and mobile medical devices the FDA seeks to standardize.

The FDA is also working with Xcertia™, a stakeholder organization formed initially by the American Heart Association, American Medical Association, Health Information and Management System Society (HIMSS), the Digital Health Group (DHX), and others in the mHealth industry to establish guidelines regarding mobile app security, privacy, content quality, operability, clinical efficacy, and ease of use, as summarized online at xcertia.org/the-guidelines/. Xcertia and the FDA hope to bring order in mHealth markets, where to date there has been inadequate oversight and standardization of mHealth products to guide consumers and caregivers.

## Asthma App Issues

### Validated Outcome Measures

Interactive asthma apps often employ symptom score tests to assess the status of a user’s disease, the results of which may inform patients how to respond to asthma exacerbations. To guarantee the quality of an app’s clinical assessments and possible recommendations, however, validated rhinitis and asthma outcomes measures are an absolute necessity.

**Fig. 2:**
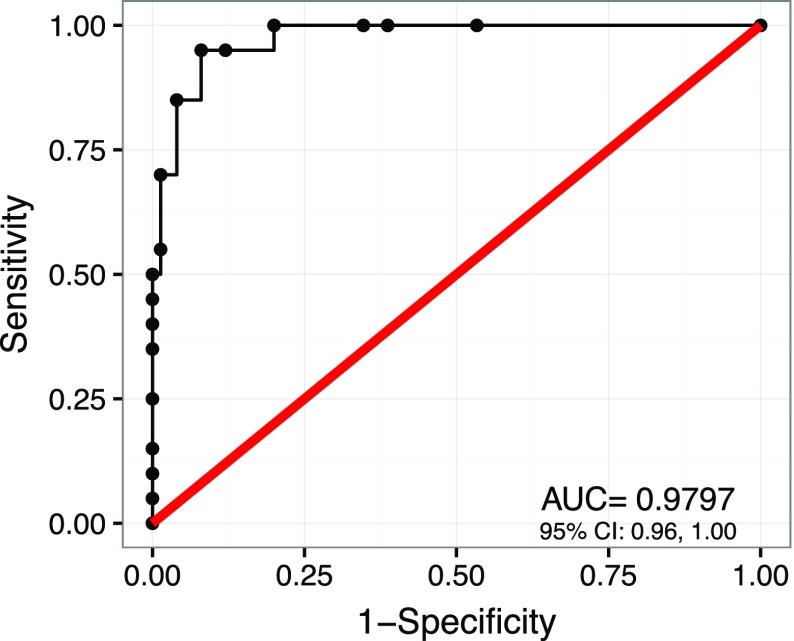
ROC curve: mobile asthma severity test vs. asthma control test. Receiver operating characteristic (ROC) curve confirming that the MAST® test and ACT® test are clinically equivalent (Kagen 2014)

mHealth apps that do not include validated outcomes measures offer nothing more than entertainment to patients and their clinicians; one cannot accurately monitor patients’ symptoms in real time without such proven instruments. The Asthma Control Test (ACT®) and Asthma Control Questionnaire (ACQ) are corporate-owned validated outcome measures employed by several mobile asthma apps [22–23]. Their owners, however, may request payments from app developers and medical clinics for the use of their intellectual property at any time, causing experienced investigators to encourage development and validation of new asthma and rhinitis questionnaires to be placed in the public domain [24]. The Mobile Asthma Severity Test (MAST® test) was developed and validated to address this issue and was shown to be equivalent to the ACT in an adult asthma population (Fig. 2) [25].

### Useless Peak Flow Tests

For decades, clinicians depended upon patient-reported peak expiratory flow (PEF) results to diagnose and treat adult and pediatric asthma patients. Even today, many primary care physicians, professional medical organizations, and school nurses promote using peak flow measurements in their Asthma Action Plans—even though peer-reviewed, well-controlled scientific publications have proven that peak flows are effort dependent, inaccurate, and nearly useless in the diagnosis and treatment of asthma.

In 1999, Brand published evidence that should have brought an end to the use of peak flow determinations when writing, “During treatment with inhaled corticosteroids the changes in PEF variation over time show poor concordance with changes in other parameters of asthma severity. When only PEF is monitored, clinically relevant deteriorations in symptoms, FEV-1 and PD20 may be missed. This suggests that home recording of PEF alone may not be sufficient to monitor asthma severity reliably in children.” [26]

There is little to no role for using peak flow data in caring for asthma patients, as summarized by Kamps: “Current asthma guidelines focus on self-management by the patient, in which monitoring of peak flow plays an important role. To be able to participate in self-management, the patient must be educated rigorously on pathophysiological mechanisms of the disease, principles of treatment, correct inhalation technique, treatment goals and the action to take when symptoms or peak flow worsen. This is a time-consuming but important and worthwhile task. The pivotal role of home peak flow monitoring in asthma self-management is based on the assumptions that peak flow variation is a useful measure of disease activity and that peak flow diaries are kept reliably by patients. There is now evidence that neither of these assumptions are true. Self-management plans based on education alone are just as effective as those incorporating peak flow monitoring. Education, therefore, is the most important component of asthma self-management, and home peak flow monitoring is not needed in the majority of asthmatic children.” [27]

Brusasco similarly stated, “In conclusion, no evidence has so far been provided to justify the inclusion of PEF measurement in asthma management plans. This recommendation should therefore be removed unless it can be shown that improving the accuracy of peak flow meters also improves compliance and clinical outcomes.” [28]

Based on these and other findings, the use of peak flow information in asthma self-management programs and mobile applications is a disservice to patients, their families, and caregivers.

### Asthma Action Plans

Controversy regarding the use of asthma action plans has existed since the concept of self-management of asthma was introduced, primarily because of reliance on inaccurate and untrustworthy patient-performed peak flow measurements. Not only are peak flow test results unreliable, they fail to correlate with clinical outcomes following asthma exacerbations [29, 30]. As Kamps and others have established, better outcomes are achieved by educating patients and their family members about a subject’s asthma triggers and patient-specific treatments, demonstrating proper inhaler techniques and monitoring patients using validated symptom score tests such as the ACT® and MAST® tests. Simply put, peak flow features do not belong in mobile asthma apps.

Some physicians, however, continue to encourage patients, school nurses, and teachers to rely on peak flow test information when creating asthma action plans, to determine when to administer a rescue inhaler or call for emergency assistance [31–33]. Whatever treatment algorithm one chooses, asthma action plans should not be based on peak flow results alone, whether such plans are provided on paper handouts in the clinic or programmed into mobile asthma applications.

### Patient-Reported Outcome Measures

The most challenging issue regarding symptom scores is convincing patients to record them routinely. Patients are understandably less motivated to record their symptoms when asymptomatic, making it difficult for clinicians to document the effectiveness of specific treatments to insurers, employers, government agencies, and other sponsors.

Two techniques known to motivate non-compliant patients are reward and punishment. Rewards may include discount coupons for products or services in collaboration with other businesses, (e.g., a local shoe store) and reduced insurance premiums as noted above [13]. The easiest approach is to compel patients to record their symptom scores when they arrive in the clinic before their appointments. For patients who refuse these gentle techniques, their prescription refills can be delayed until clinicians see the patient’s test scores.

### Medication Adherence

Failure to comply with asthma treatment plans has been a major cause of hospital readmissions, unfortunate asthma deaths, higher health care costs, and lower quality of life in both pediatric and adult asthma populations [34–39]. This problem has been mitigated in part by the development of electronic sensing devices that attach to inhaled respiratory medications and communicate with prescribing clinicians when inhalers are actuated. After detecting an actuation, a signal is transmitted via a Bluetooth-connected smartphone to secure Cloud-based databases accessible in real time to patients and their caregivers, enabling clinicians to monitor individual patients and an entire clinic population simultaneously.

Actuation sensors are now available worldwide, and their use has been associated with lower overall costs for asthma care and better clinical outcomes [40–56]. Three peer-reviewed sensor studies are summarized in Table 3. Sensors from Adherium (adherium.com/) and Propeller Health (www.propellerhealth.com) were assessed for their effectiveness on adherence in combination with patient education and daily delivery of app-based medication reminders to test subject’s smartphones.

**Table 3:** Respiratory inhaler sensor studies

App	Author	Study	Outcomes
Adherium (Hailie)	Foster (2014)	Study:	ACT:
		40 primary care physicians trained in asthma care and remote monitoring of asthma compare sensor users vs. non-users	No difference in ACT scores between remote sensor users receiving feedback and sensor non-user control group
			Prednisone:
		Patients:	No difference between sensor users and non-users re prednisone use (7/64 sensor users vs. 19/67 sensor non-users)
		113 asthma patients in a randomized controlled 6-month comparison study	
		Outcome measures:	Adherence:
			Adherence declined in both groups, but was greater in sensor users than non-users (73% ± 26% vs. 28% ± 28%; *P* = < 0.001)
		ACT scores, adherence, and prednisone use	Conclusion:
			Remote sensor notifications increase patient compliance with inhaled asthma medication
Adherium (Hailie)	Morton (2016)	Study:	ACQ + FEV-1:
		Randomized, controlled 12-month study of sensor users and non-users	No differences observed in ACQ or FEV-1 between sensor users and sensor non-users re symptom scores or FEV-1
		Patients:	
		89 poorly controlled pediatric asthma patients (ACQ > 1.5): 47 sensor users and 42 sensor non-users	Adherence:
			Adherence declined in both groups, but sensor users were more compliant than non-users
		Outcome measures:	Prednisone:
		ACQ, FEV-1, hospitalization, unplanned office visits, and prednisone use	Sensor users were 53% less likely to need prednisone than sensor non-users
			Hospitalization:
			Rates of hospitalization per 100 child days were significantly less for sensor users than non-users
			Conclusions:
			Using remote sensors, patient reminders, and feedback decreased prednisone use and hospitalization rates in children with poorly controlled asthma
Propeller Health	Merchant (2017)	Study:	Hospitalization:
		5-year retrospective analysis of resource utilization rates per 100 patient-years by 507 asthma patients before and after using a remote sensor and app-based feedback	79% decline in rate of use/100 patient-years
		Patients:	
		507 asthma patient records reviewed pre + post remote sensor use	
		Outcome measures:	
		Rates of hospitalization, emergency room visits, and outpatient office visits for asthma	Emergency room:
			57% decline rate of use/100 patient-years
			Outpatient asthma visits:
			41% increase office visits for asthma
			Conclusions:
			Mobile app-based sensors and feedback was associated with increased office visits for asthma and reduced asthma-related resource utilization

Foster [53], Morton [55], and Merchant [56] conducted controlled studies in adult and pediatric asthma patients comparing sensor users with non-users. These studies demonstrated significant clinical and economic benefits associated with actuation sensor use, even though adherence in both sensor user and non-user groups declined over time. Interestingly, Foster found no difference in ACT scores between sensor users receiving feedback and sensor non-users. Use of actuation sensors, however, was associated with a significant decline in emergency room visits and asthma hospitalizations.

### Asthma App Quality

Until recently, app quality was difficult to determine objectively, primarily because reliable measures of app quality, content, and usability were non-existent. Progress has been made, however, with development of the Mobile App Rating Scale (MARS) created at Queensland University of Technology [57, 58]. The MARS uses a five-point Likert scale to score four well-defined app categories: user engagement, functionality, esthetics, and information accuracy. Scores from each category are then summed to arrive at overall objective and subjective quality scores. (See: MARS questionnaire online).

Baptista acknowledged the MARS is a positive step forward and suggested more work remains, for MARS validation studies regarding structure and content were composed of small numbers of patients with asthma, heart failure, and obesity [59]. Thus, the science of determining app quality is an evolving science.

It may be impossible to create a single measurement tool for app quality in all circumstances for several reasons. First, patients, caregivers, payers, sponsors, and regulators will have different opinions of quality, and second, digital health apps designed for one disease may not accurately reflect the quality of another mHealth app designed for a different illness. For example, allergic rhinitis and asthma require different validated outcomes measures.

With this limitation in mind, MARS scores for three leading interactive mobile asthma apps are presented in Table 4. While Hailie, Propeller Health, and KagenAir asthma apps are unique, their overall MARS quality scores are comparable in all four categories. Likewise, MARS scores for two popular standalone asthma apps, Asthma Storylines and AsthmaMD, are similar in overall quality, but the subjective quality of Asthma Storylines is superior to AsthmaMD. (See: MARS app quality scores online).

**Table 4:** Mobile Application Rating Scale (MARS) scores

Interactive asthma apps	AsthmaMD	Asthma Storylines	Hailie	KagenAir	Propeller Health
Engagement	3.00	4.40	4.40	4.60	4.40
Functionality	4.50	4.75	5.00	5.00	4.50
Esthetics	4.50	4.50	5.00	5.00	4.50
Information	4.17	4.33	3.33	4.71	5.00
App quality MARS mean	4.04	4.50	4.43	4.83	4.60
Subjective quality MARS mean	2.00	3.75	4.75	4.75	4.60

## Standalone Asthma Apps

Standalone asthma apps allow users to enter, store, or collect information about themselves or other people without being able to send such data to others. Standalone apps are also less costly to design, create, and maintain than their interactive counterparts and are frequently downloaded from the App Store and Google Play Store. The majority of standalone allergy apps, however, are marketing tools for over-the-counter antihistamine products or promote independent allergy clinics. Although not discussed in this review, 12 standalone apps available are listed in Table 5, the most popular being Asthma Storylines and AsthmaMD.

**Table 5:** Standalone mHealth allergy and asthma apps (2018)

App	Owner	Website	Notes
AccuPollen	Len Bielory M.D.	www.nynjpollen.com	NAB pollen information
Allergy 123	Preventive Health Diagnostics, Inc.	http://www.allergy123.com	Remote allergy practice sales and marketing
Allergy Alert	IQVIA	www.iqvia.com/	Commercial sales
AllergyCast	Johnson+Johnson	www.zyrtec.com/	Commercial sales—Zyrtec
Allergy Pollen Count	Ghassan Safadi, M	http://www.allergyclinicohio.com	Clinic business app
AP pal	Allergy Partners	https://www.allergypartners.com/introducing-appal/	Clinic business app
Asthma & Me	Anthem, Inc.	mydiversepatients.com/	Must be a patient
Change 6401	Cincinnati Children’s Hospital Med. Center	https://www.cincinnatichildrens.org/	
Kiss My Asthma	Univ. of Sydney	kissmyasthma.org.au/	For patients in Australia
MyAsthma	GlaxoSmithKline, PLC	https://www.gsk.com /	Commercial sales, ACT®
My Asthma Pal	Children’s Med. Center Dallas	www.childrens.com/	Clinic business app, ACT®
WebMD	KKR & Co Inc.	www.kkr.com	Commercial sales Education

Asthma Storylines is sponsored by the Allergy & Asthma Network, a non-profit organization active nationwide in public education and government affairs. The Asthma Storylines app is free to consumers, allows users-patients to input and store medication and inhaler usage data, locate board-certified specialists in the American College of Allergy, Asthma and Immunology and receive secure notifications and daily medication reminders. Storylines also has an asthma action plan that is dependent on patient-performed peak flow test results and the ACT® symptom score test.

Although the AsthmaMD app is free and allows users-patients to input and store their personal health information, it is less functional than the Asthma Storylines app primarily because it uses an unverified asthma action plan.

## Interactive Asthma Apps

Hailie, KagenAir, and Propeller interactive asthma apps offer the greatest number of features and mobile services as summarized in Table 6.

**Table 6:** Features in mobile asthma apps—2018

27 Features	Asthma Storylines	AsthmaMD	KagenAir	Propeller	Hailie
Classification	Standalone	Standalone	Interactive	Interactive	Interactive
Cost					
Free to user	Yes	Yes	Yes	Yes	Yes
Clinic pays for access	–	–	Yes	Yes	Yes
Adherence					
User input data	Yes	Yes	Yes	Yes	Yes
Automatic input of data	–	–	–	Yes	Yes
Validated symptom score tests					
Public domain	–	–	Yes	–	–
Non-public domain	Yes	–	–	Yes	–
Alerts					
Inhaler use—user inputs	Yes	–	Yes	Yes	–
Inhaler use—auto	–	–	–	Yes	Yes
Asthma flares	–	–	Yes	Yes	Pending
Forecast symptoms	–	–	Yes	Yes	Pending
Trigger identification					
Artificial intelligence	–	–	Yes	Yes	Pending
Measurements					
FEV-1 user input data	–	Yes	Yes	Yes	–
FeNO user input data	–	–	Yes	–	–
Peak flow user inputs	Yes	Yes	–	Yes	Yes
Hypersensitivity test	–	–	Yes	–	–
Management					
Asthma action plan	Yes	Yes	–	Yes	Pending
Emergency instructions	–	Yes	–	Yes	Pending
Prednisone use user input	Yes	–	Yes	Yes	Pending
Population mgmt	–	–	Yes	Yes	Pending
ER + urgent visits	–	–	Yes	Yes	Pending
Communications					
Secure notifications	Yes	–	Yes	Yes	Yes
Telemedicine	–	–	Yes	–	–
Educational content	Yes	Yes	Yes	Yes	Yes
Monitor aeroallergens					
Local pollens + molds	–	–	Yes	–	Pending
User location					
GPS	–	–	Yes	Yes	Yes
Connect to caregivers					
Bd-certified allergists	Yes	–	Yes	–	–
Compare symptoms w/					
Weather + pollution	–	–	Yes	Yes	Pending
Feature score	10	7	20	21	9

**Fig. 3:**
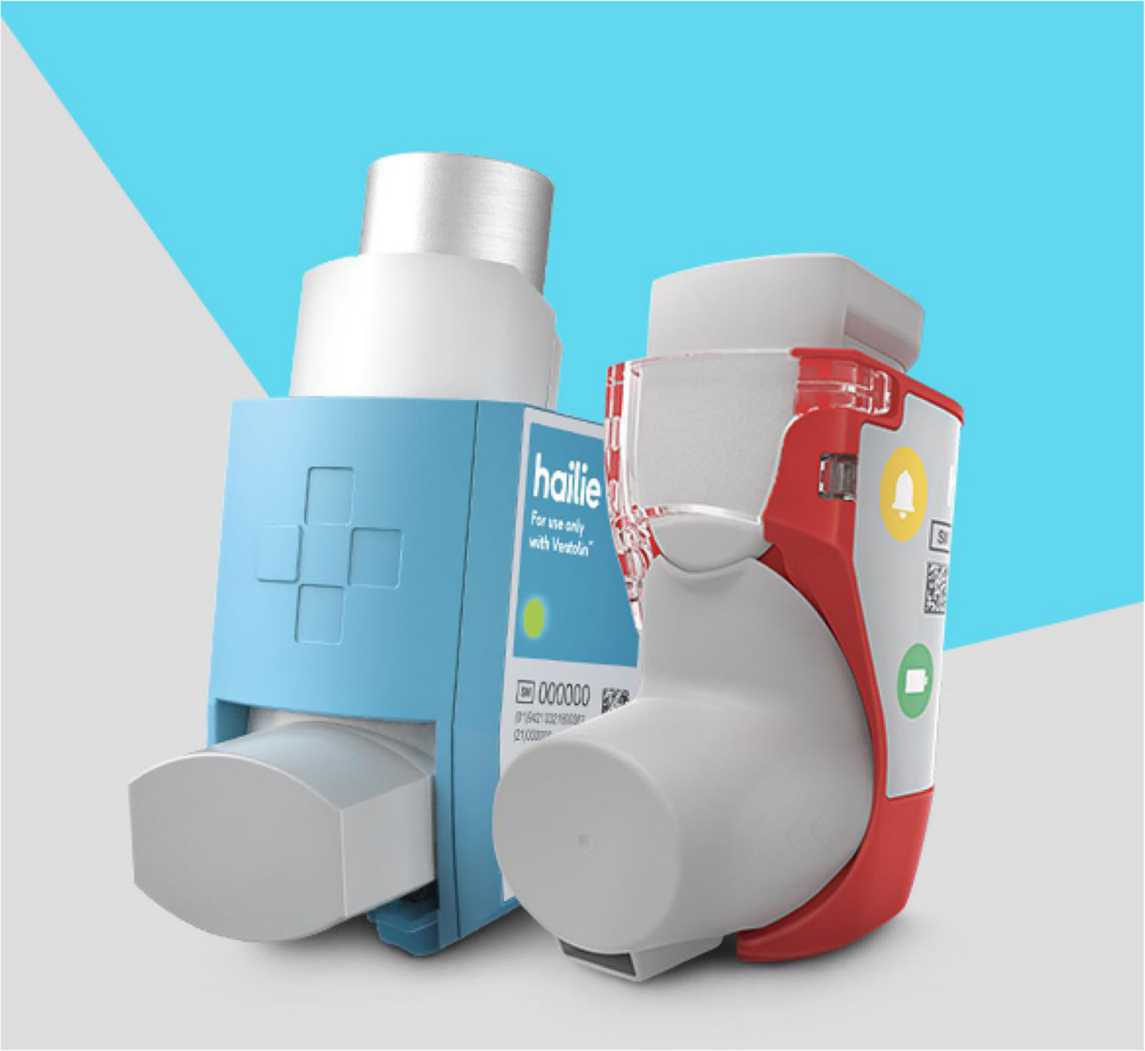
Hailie sensor

The Hailie actuation sensor is available for purchase by consumers online and/or free to patients from their health insurance benefits. It offers nine essential features, including use of its patented sensing devices that attach to most inhaled medications available in the USA (Fig. 3). Hailie allows users to input and share their peak flow test results, enabling subscribing caregivers to monitor their patients’ adherence remotely in real time via a secure web-based portal. Nine additional features are currently being programmed into the Hailie app and Cloud-based portal to enable caregivers to monitor and communicate with individual patients and entire clinic populations.

The KagenAir mobile app is free to consumers, offering 20 features for rhinitis and asthma patients and their subscribing caregivers at KagenAir.com [60]. Its features include two public domain validated symptom score tests for both rhinitis and asthma, secure delivery of notifications and medication reminders, patent-pending artificial intelligent software that identifies a user’s unique environmental triggers, connections to nearby GPS-located board-certified allergy and asthma specialists, a proprietary hypersensitivity test to determine the degree of one’s autonomic nervous system reactivity, and live telemedicine communications between users and their chosen physicians. Unlike Propeller and Hailie applications, KagenAir does not use peak flow information in creating asthma action plans.

Associated with the KagenAir application is an iPhone-based mobile application that accurately measures smooth muscle speeds of contraction during the pupillary light reflex, the Sensitometer® test at Sensitometer.com [61, 62]. Studies are underway to determine if this mobile app can identify patients with hyperreactive airways (e.g., asthma) as pupillary smooth muscles reflect smooth muscles throughout the body and autonomic nervous system function [63].

**Fig. 4:**
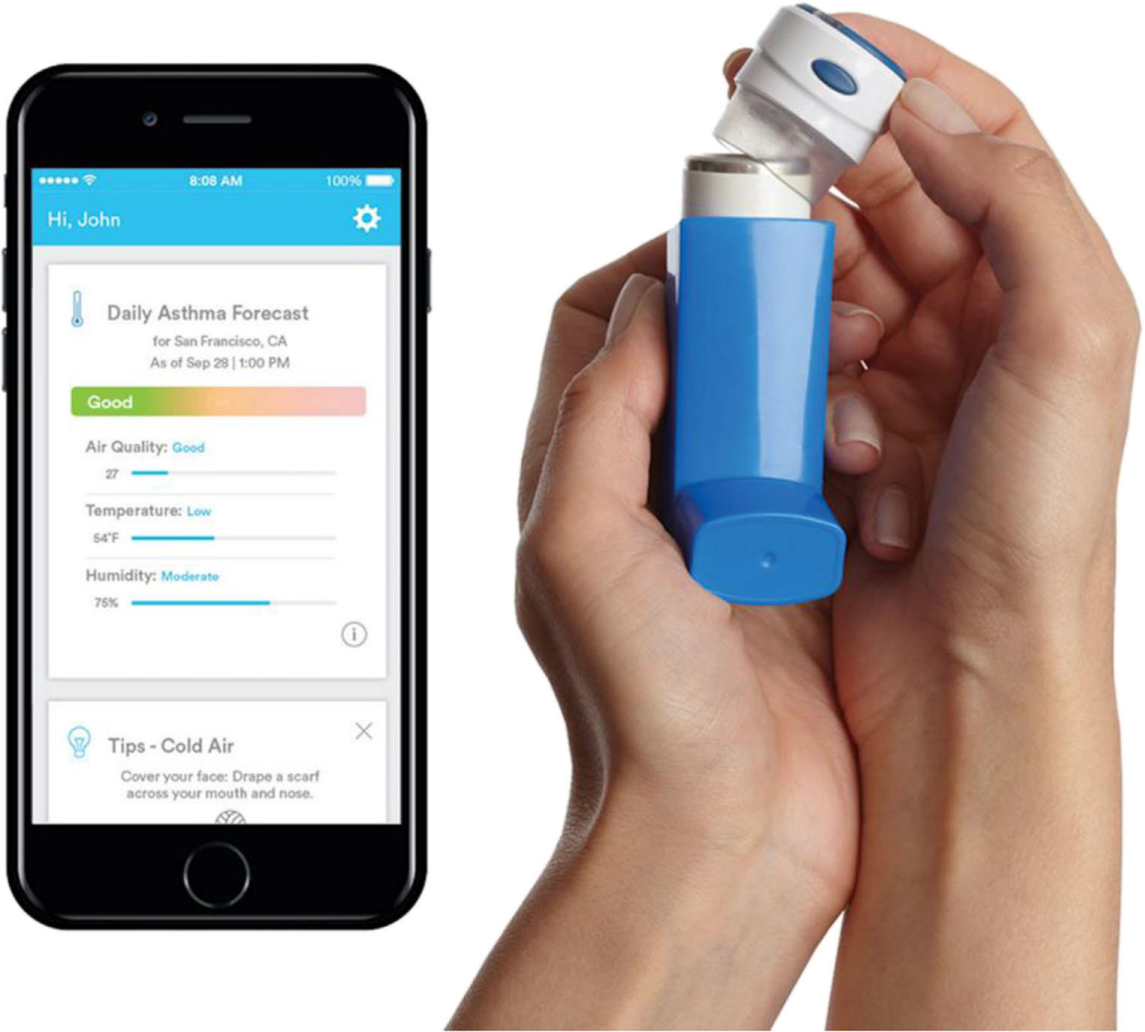
Propeller sensor

The Propeller mobile asthma app is well established in the USA, providing 18 essential features to subscribing patients and clinics including automatic recording of inhaler actuations, an asthma action plan dependent on peak flow data, use of prescription medications, emergency room visits, real-time population oversight, and management and secure notifications between app users and caregivers (Fig. 4). It also sends patients an alert to contact their caregivers when rescue inhaler use increases, provides educational materials, and identifies correlations between environmental triggers and user symptoms. Propeller does not offer telemedicine services, nor does it use a public domain validated asthma symptom score measure.

App performance scores reflect the overall acceptability of mobile applications in one’s personal life and clinic workflows with scores ranging between 0 and 10, as presented in Table 7. The more comprehensive services offered by Propeller, Hailie, and KagenAir enterprise applications are better suited for integration into clinic settings than standalone products.

**Table 7:** Mobile asthma app performance scores

App performance	Asthma Storylines	AsthmaMD	KagenAir	Propeller Health	Hailie
Classification	Standalone	Standalone	Interactive	Interactive	Interactive
1. Easy to use	Yes	Yes	Yes	Yes	Yes
2. Reliable	Yes	No	Yes	Yes	Yes
3. Easy clinic integration	No	No	Easy	Easy	Easy
4. Staff acceptance	–	–	Yes	Yes	N/A
5. Privacy + security	Yes	Yes	Yes	Yes	Yes
6. Design: bad-simple-good-excellent	Good	Simple	Excellent	Excellent	Excellent
7. User feedback	Yes	No	Feedback	Feedback	Feedback
8. Caregiver evaluations	No	No	No	Yes	Yes
9. Support + updates	Yes	No	Yes	Yes	Yes
10. Language	English	English	English	Eng + 11 languages	Eng, Fr Span Dutch
Total score [0–10]	5	2	9	10	9

CareTRx is an iPhone and Android mobile app designed to monitor patients with asthma and other respiratory disorders (www.caretrx.com/), but as it requires patients-users to obtain it via a medical clinic program, we were unable to consider it in this review.

## Mobile Health Implementation

How easily can mobile health features and services be incorporated into one’s clinical practice? Where does one begin? The journey starts with the understanding that mHealth connects patients seamlessly with their caregivers via mobile smartphones and other digital devices, including secure video communications and health information recorded from wearable devices.

Before investing in mobile health technology, the authors suggest answering these questions about your current clinic workflow and practice patterns.

1. Who will be responsible for reviewing federal and state regulations, and existing insurance plan coverages for mHealth services?
2. Who will perform the telemedicine video-office visits, and what training will they require? Where will you conduct these video visits?
3. What mobile health services are you willing to offer (e.g., telemedicine, inhaler adherence monitoring)?
4. Who will be your technical and clinical decisionmakers?
5. What are your technology acquisition and maintenance costs?
6. Will the vendor’s equipment and software be compatible with your current electronic health record software?
7. How will you integrate mHealth services and telemedicine into your clinic workflow?
8. Who will schedule your telemedicine visits and invoice your patients?
9. How will you measure the value of your services to payers?
10. How will you measure clinical outcomes?
11. Have you evaluated your competitor’s services and prices, and developed a winning marketing plan?

There are many choices to make. Take your time and choose wisely knowing the digital health marketplace will be ever-changing and improving communications between patients and their caregivers.

## Summary

Interactive mobile asthma apps are valuable assets for patients and caregivers alike, for they offer immediate communications between patients and those responsible for providing for their needs. Primary care and specialty clinicians interested in introducing digital health apps into their practices will soon have more choices, for Apple and other electronic medical record software companies are investing heavily in the mobile medical marketplace, guaranteeing personal health information and access to care will always be immediately available in one’s *digital hand*.

## Electronic Supplementary Material

ESM 1 (PDF 138 kb)

ESM 2 (PDF 138 kb)

ESM 3 (PDF 138 kb)

ESM 4 (PDF 139 kb)

ESM 5 (PDF 138 kb)

ESM 6 (PDF 261 kb)

## Abbreviations

ACT®: Asthma Control Test®
App: Software application within mobile communication devices
AST: Allergy Severity Test
BML: Build-measure-learn agile software development process
FEV-1: Forced expiratory volume in 1 s
FTC: US Federal Trade Commission
HIMSS: Health Information and Management System Society
Interactive app: An app that receives and sends information in real time or asynchronously
MAST® test: Mobile Asthma Severity Test®
mHealth: Mobile digital communication devices and software applications engaged in human health
Standalone app: Users may enter, store, and/or collect information about themselves, without the ability to send such data to others
