# Supplementary material for: Asthma and Allergy Mobile Apps in 2018
Source: Curr Allergy Asthma Rep. 2019 Feb 2;19(1):6. doi: 10.1007/s11882-019-0840-z (PMC6394463; doi:10.1007/s11882-019-0840-z)
Supplement: Supplementary file 1 — (PDF 138 kb) [file 11882_2019_840_MOESM1_ESM.pdf]

Mobile Application Rating Scale (MARS) App Classification

The Classification section is used to collect descriptive and technical information about the app. Please review the app description in iTunes / Google Play to access this information.

App Name: Hailie **Date:** 7-22-2018

Rating this version: **Rating all versions:** ____________________

Developer: Adherium Ltd (NZ)

N ratings this version:____________________ **N ratings all versions:** ____________________

Version: 6.0.1.5 **Last update:** 3 weeks ago.

Cost - basic version: Free to Consumer **Cost - upgrade version:** Free

Platform: X iPhone X iPad X Android

Brief description: Hailie consists of an monitor that transmits inhaler usage to a Cloud-based portal, displays it to Users and selected Caregivers.

____

Focus: what the app targets
(select all that apply)

- □ Increase Happiness/Well-being
- □ Mindfulness/Meditation/Relaxation
- □ Reduce negative emotions
- □ Depression
- □ Anxiety/Stress
- □ Anger
- □ Behaviour Change
- □ Alcohol /Substance Use
- □ Goal Setting
- □ Entertainment
- □ Relationships
- □ Physical health
- X Asthma Monitoring

Theoretical background/Strategies
(all that apply)

- □ Assessment
- □ Feedback
- □ Information/Education
- □ Monitoring/Tracking
- □ Goal setting
- □ Advice /Tips /Strategies /Skills training
- □ CBT - Behavioural (positive events)
- □ CBT – Cognitive (thought challenging)
- □ ACT - Acceptance commitment therapy
- □ Mindfulness/Meditation
- □ Relaxation
- □ Gratitude
- □ Strengths based

Affiliations:

X Commercial

Age group (all that apply)

- □ Children (under 12)
- □ Adolescents (13-17)
- □ Young Adults (18-25)
- □ Adults
- □ General

Technical aspects of app (all that apply)

- □ Allows sharing (Facebook, Twitter, etc.)
- □ Has an app community
- □ Allows password-protection
- □ Requires login
- □ Sends reminders
- □ Needs web access to function

App Quality Ratings

The Rating scale assesses app quality on four dimensions. All items are rated on a 5-point scale from "1.Inadequate" to "5.Excellent". Circle the number that most accurately represents the quality of the app component you are rating. Please use the descriptors provided for each response category.

SECTION A

Engagement – fun, interesting, customisable, interactive (e.g. sends alerts, messages, reminders, feedback, enables sharing), well-targeted to audience

1. **Entertainment: Is the app fun/entertaining to use? Does it use any strategies to increase engagement through entertainment (e.g. through gamification)?**

   1 Dull, not fun or entertaining at all
   2 Mostly boring
   3 OK, fun enough to entertain user for a brief time (< 5 minutes)
   4 Moderately fun and entertaining, would entertain user for some time (5-10 minutes total)
   5 Highly entertaining and fun, would stimulate repeat use

2. **Interest: Is the app interesting to use? Does it use any strategies to increase engagement by presenting its content in an interesting way?**

   1 Not interesting at all
   2 Mostly uninteresting
   3 OK, neither interesting nor uninteresting; would engage user for a brief time (< 5 minutes)
   4 Moderately interesting; would engage user for some time (5-10 minutes total)
   5 Very interesting, would engage user in repeat use

3. **Customisation: Does it provide/retain all necessary settings/preferences for apps features (e.g. sound, content, notifications, etc.)?**

   1 Does not allow any customisation or requires setting to be input every time
   2 Allows insufficient customisation limiting functions
   3 Allows basic customisation to function adequately
   4 Allows numerous options for customisation
   5 Allows complete tailoring to the individual's characteristics/preferences, retains all settings

4. **Interactivity: Does it allow user input, provide feedback, contain prompts (reminders, sharing options, notifications, etc.)? Note: these functions need to be customisable and not overwhelming in order to be perfect.**

   1 No interactive features and/or no response to user interaction
   2 Insufficient interactivity, or feedback, or user input options, limiting functions
   3 Basic interactive features to function adequately
   4 Offers a variety of interactive features/feedback/user input options
   5 Very high level of responsiveness through interactive features/feedback/user input options

5. **Target group: Is the app content (visual information, language, design) appropriate for your target audience?**

   1 Completely inappropriate/unclear/confusing
   2 Mostly inappropriate/unclear/confusing
   3 Acceptable but not targeted. May be inappropriate/unclear/confusing
   4 Well-targeted, with negligible issues
   5 Perfectly targeted, no issues found

A. Engagement mean score = 22 / 5 = 4.40

SECTION B

Functionality – app functioning, easy to learn, navigation, flow logic, and gestural design of app

6. Performance: How accurately/fast do the app features (functions) and components (buttons/menus) work?

1 App is broken; no/insufficient/inaccurate response (e.g. crashes/bugs/broken features, etc.)
2 Some functions work, but lagging or contains major technical problems
3 App works overall. Some technical problems need fixing/Slow at times
4 Mostly functional with minor/negligible problems
5 Perfect/timely response; no technical bugs found/contains a 'loading time left' indicator

7. Ease of use: How easy is it to learn how to use the app; how clear are the menu labels/icons and instructions?

1 No/limited instructions; menu labels/icons are confusing; complicated
2 Useable after a lot of time/effort
3 Useable after some time/effort
4 Easy to learn how to use the app (or has clear instructions)
5 Able to use app immediately; intuitive; simple

8. Navigation: Is moving between screens logical/accurate/appropriate/ uninterrupted; are all necessary screen links present?

1 Different sections within the app seem logically disconnected and random/confusing/navigation is difficult
2 Usable after a lot of time/effort
3 Usable after some time/effort
4 Easy to use or missing a negligible link
5 Perfectly logical, easy, clear and intuitive screen flow throughout, or offers shortcuts

9. Gestural design: Are interactions (taps/swipes/pinches/scrolls) consistent and intuitive across all components/screens?

1 Completely inconsistent/confusing
2 Often inconsistent/confusing
3 OK with some inconsistencies/confusing elements
4 Mostly consistent/intuitive with negligible problems
5 Perfectly consistent and intuitive

B. Functionality mean score = 20 / 4 = 5.00

SECTION C

Aesthetics – graphic design, overall visual appeal, colour scheme, and stylistic consistency

10. Layout: Is arrangement and size of buttons/icons/menus/content on the screen appropriate or zoomable if needed?

1 Very bad design, cluttered, some options impossible to select/locate/see/read device display not optimised
2 Bad design, random, unclear, some options difficult to select/locate/see/read
3 Satisfactory, few problems with selecting/locating/seeing/reading items or with minor screen-size problems
4 Mostly clear, able to select/locate/see/read items
5 Professional, simple, clear, orderly, logically organised, device display optimised. Every design component has a purposeGraphics: How high is the quality/resolution of graphics used for buttons/icons/menus/content?

1 Graphics appear amateur, very poor visual design - disproportionate, completely stylistically inconsistent
2 Low quality/low resolution graphics; low quality visual design – disproportionate, stylistically inconsistent
3 Moderate quality graphics and visual design (generally consistent in style)
4 High quality/resolution graphics and visual design – mostly proportionate, stylistically consistent
5 Very high quality/resolution graphics and visual design - proportionate, stylistically consistent throughout

11. Visual appeal: How good does the app look?

1 No visual appeal, unpleasant to look at, poorly designed, clashing/mismatched colours
2 Little visual appeal – poorly designed, bad use of colour, visually boring
3 Some visual appeal – average, neither pleasant, nor unpleasant
4 High level of visual appeal – seamless graphics – consistent and professionally designed
5 As above + very attractive, memorable, stands out; use of colour enhances app features/menus

C. Aesthetics mean score = 10 / 2 = 5.00

SECTION D

Information – Contains high quality information (e.g. text, feedback, measures, references) from a credible source. Select N/A if the app component is irrelevant.

12. Accuracy of app description (in app store): Does app contain what is described?

1 Misleading. App does not contain the described components/functions. Or has no description
2 Inaccurate. App contains very few of the described components/functions
3 OK. App contains some of the described components/functions
4 Accurate. App contains most of the described components/functions
5 Highly accurate description of the app components/functions

13. Goals: Does app have specific, measurable and achievable goals (specified in app store description or within the app itself)?

N/A Description does not list goals, or app goals are irrelevant to research goal (e.g. using a game for educational purposes)
1 App has no chance of achieving its stated goals
2 Description lists some goals, but app has very little chance of achieving them
3 OK. App has clear goals, which may be achievable.
4 App has clearly specified goals, which are measurable and achievable
5 App has specific and measurable goals, which are highly likely to be achieved.

14. Quality of information: Is app content correct, well written, and relevant to the goal/topic of the app?

N/A There is no information within the app
1 Irrelevant/inappropriate/incoherent/incorrect
2 Poor. Barely relevant/appropriate/coherent/may be incorrect
3 Moderately relevant/appropriate/coherent/and appears correct
4 Relevant/appropriate/coherent/correct
5 Highly relevant, appropriate, coherent, and correct

15. Quantity of information: Is the extent coverage within the scope of the app; and comprehensive but concise?

N/A There is no information within the app
1 Minimal or overwhelming
2 Insufficient or possibly overwhelming
3 OK but not comprehensive or concise
4 Offers a broad range of information, has some gaps or unnecessary detail; or has no links to more information and resources
5 Comprehensive and concise; contains links to more information and resources

16. Visual information: Is visual explanation of concepts – through charts/graphs/images/videos, etc. – clear, logical, correct?

N/A There is no visual information within the app (e.g. it only contains audio, or text)
1 Completely unclear/confusing/wrong or necessary but missing
2 Mostly unclear/confusing/wrong
3 OK but often unclear/confusing/wrong
4 Mostly clear/logical/correct with negligible issues
5 Perfectly clear/logical/correct

17. Credibility: Does the app come from a legitimate source (specified in app store description or within the app itself)? N/A

1 Source identified but legitimacy/trustworthiness of source is questionable (e.g. commercial business with vested interest)
2 Appears to come from a legitimate source, but it cannot be verified (e.g. has no webpage)
3 Developed by small NGO/institution (hospital/centre, etc.) /specialised commercial business, funding body
4 Developed by government, university or as above but larger in scale
5 Developed using nationally competitive government or research funding (e.g. Australian Research Council, NHMRC)

18. Evidence base: Has the app been trialled/tested; must be verified by evidence (in published scientific literature)?

N/A The app has not been trialled/tested
1 The evidence suggests the app does not work
2 App has been trialled (e.g., acceptability, usability, satisfaction ratings) and has partially positive outcomes in studies that are not randomised controlled trials (RCTs), or there is little or no contradictory evidence.
3 App has been trialled (e.g., acceptability, usability, satisfaction ratings) and has positive outcomes in studies that are not RCTs, and there is no contradictory evidence.
4 App has been trialled and outcome tested in 1-2 RCTs indicating positive results
5 App has been trialled and outcome tested in $\geq$ 3 high quality RCTs indicating positive results

D. Information mean score = 20 / 6 = 3.33

* Exclude questions rated as “N/A” from the mean score calculation.

App subjective quality

SECTION E

19. Would you recommend this app to people who might benefit from it?

1	**Not at all**	I would not recommend this app to anyone
2		There are very few people I would recommend this app to
3	**Maybe**	There are several people whom I would recommend it to
4		There are many people I would recommend this app to
5	**Definitely**	I would recommend this app to everyone

20. How many times do you think you would use this app in the next 12 months if it was relevant to you?

1 **None**
2 1-2
3 3-10
4 10-50
5 >50

21. Would you pay for this app?

1 No
3 Maybe
5 Yes

22. What is your overall star rating of the app?

1	"	One of the worst apps I've used
2	""	
3	"""	Average
4	""""	
5	"""""	One of the best apps I've used

Scoring

App quality scores for

SECTION

A: Engagement Mean Score = 4.40

B: Functionality Mean Score = 5.00

C: Aesthetics Mean Score = 5.00

D: Information Mean Score = 3.33

App quality mean Score = 4.43

App subjective quality Score = 19 / 4 = 4.75

App-specific

These added items can be adjusted and used to assess the perceived impact of the app on the user's knowledge, attitudes, intentions to change as well as the likelihood of actual change in the target health behaviour.

SECTION F

1. **Awareness: This app is likely to increase awareness of the importance of addressing (insert target health behavior)**

Strongly disagree				Strongly Agree
1	2	3	4	5

2. **Knowledge: This app is likely to increase knowledge/understanding of [insert target health behaviour]**

Strongly disagree				Strongly Agree
1	2	3	4	5

3. **Attitudes: This app is likely to change attitudes toward improving [insert target health behaviour]**

Strongly disagree				Strongly Agree
1	2	3	4	5

4. **Intention to change: This app is likely to increase intentions/motivation to address [insert target health behaviour]**

Strongly disagree				Strongly Agree
1	2	3	4	5

5. **Help seeking: Use of this app is likely to encourage further help seeking for [insert target health behaviour] (if it's required)**

Strongly disagree				Strongly Agree
1	2	3	4	5

6. **Behaviour change: Use of this app is likely increase/decrease [insert target health behaviour]**

Strongly disagree				Strongly Agree
1	2	3	4	5
